# 

**Published:** 1888-05

**Authors:** 


### CONTENTS.

COMMUNICATIONS.

Astiology of Irregularities of the Jaws and Teeth........ ........ ..... 213
Implantation with Clinical Demonstration.................................. 233
A Case in Office Practice ............................................... 238
Immediate Root Filling.................................................. 242

PROCEEDINGS.

Forty-Fourth Annual Meeting of the Mississippi Valley Association of Dental Surgeons............................................. 245
First Annual Meeting of the Stockholders of the Ohio Dental College ................................................................ 253

SELECTIONS.

Medical Missionaries .................................................. 257
Contribution to the Etiology of Neuralgia................................ 258
SALMAGUNDI ............................................................ 261

EDITORIAL.

Obituary............................................................ 263
Bibliographical....................................................... 264

